# The Individual-Level and Community-Level Social Determinants of Burn Injuries: A Single-Institution Study From the Southwestern United States

**DOI:** 10.1093/jbcr/irae131

**Published:** 2024-07-06

**Authors:** Samuel Cohler, Henry Krasner, Kavita Batra, Syed Saquib

**Affiliations:** Kirk Kerkorian School of Medicine at UNLV, University of Nevada Las Vegas, Las Vegas, NV 89106, USA; Kirk Kerkorian School of Medicine at UNLV, University of Nevada Las Vegas, Las Vegas, NV 89106, USA; Kirk Kerkorian School of Medicine at UNLV, University of Nevada Las Vegas, Las Vegas, NV 89106, USA; Department of Medical Education, Kirk Kerkorian School of Medicine at UNLV, University of Nevada Las Vegas, Las Vegas, NV 89106, USA; Department of Surgery, Kirk Kerkorian School of Medicine at University of Nevada Las Vegas, Las Vegas, NV 89106, USA; University Medical Center Lions Burn Care Center, Las Vegas, NV 89106, USA

**Keywords:** burn injuries, socioeconomic status, demographic factors

## Abstract

Burn injuries are a significant public health concern, causing life-threatening complications and substantial hospitalization costs for patients. It has been shown that burn injuries may affect individuals differently based on demographic factors and socioeconomic status, among other variables. In the Southwestern United States with high ambient temperatures, specific burn etiologies, such as pavement burns, may pose a disproportionately high risk for disadvantaged communities and homeless individuals. This study uniquely explores burn injuries in relation to patients’ socioeconomic status in Las Vegas, Nevada by using the Distressed Community Index to quantify socioeconomic status utilizing individual-level and community-level indicators. This single-institution and retrospective study collected data from all patients admitted to a burn center located in Las Vegas. Data were analyzed through Chi-square, one-way ANOVA, and post-hoc analysis with Tukey’s test. Patients residing in distressed communities contributed to the greatest number of burn injuries; however, there was a lack of significant association between socioeconomic status and burn injury (*P* = 202). Additionally, specific burn etiologies and demographic characteristics were associated with variations in burn patient hospital course, complications, resources utilized and outcomes. Distressed patients were significantly associated with public insurance (*P* < 0.001), and public insurance users were associated with pavement burns—one of the most severe burn injuries (*P* < 0.001). This study emphasizes the importance of developing comprehensive burn prevention resources tailored to vulnerable populations, especially in regions with increased incidence of severe burn injuries, in order to reduce burn burden and mortality.

## INTRODUCTION

Burn injuries are a significant public health concern that may result in substantial medical consequences for patients. Severe burn injuries have been shown to cause numerous life-threatening complications, including hypovolemic shock and sepsis, and may require extensive long-term management for patients.^1–4^ Furthermore, according to the World Health Organization, it is estimated that 180 000 people die due to burn injuries annually.^5^ Even with interventions, critically ill burn patients who survive hospitalization are associated with decreased 5-year survival rates compared to patients with less severe burns or without burns, particularly due to trauma and psychological ramifications of their injuries.^6,7^ Burn injuries may additionally result in lengthy hospitalizations for patients.^8,9^ One US study found that length of stay (LOS) increased as total body surface area (TBSA) burned increased, with patients with >75% TBSA hospitalized for 93 days on average.^8^ It has also been demonstrated that burn injuries place a significant financial burden on patients. An Australian retrospective study from 2012 reported that the average cost for adult burn patients was over $70 000 per hospitalization, and this cost was correlated with the percentage of TBSA.^10^ Another study indicated that the annual cost of burn treatment in the United States is greater than $1 billion, not including costs of rehabilitation and disability.^11^

One factor that may influence the burden of burn injuries on patients is the specific etiology. There are various potential etiologies for burn injuries, which may vary by regional and demographic characteristics.^12–14^ Overall, thermal burns tend to be the most common causes for burn-related hospitalization, including scald and flame burn injuries.^14–17^ Yet in geographic regions including the Southwestern United States, pavement and cement burns have greater incidence.^18,19^ It has been shown that the risk of pavement burns in desert climates begins around 95°F and increases exponentially as ambient temperature rises.^12^ A study in Las Vegas, Nevada stated that asphalt temperatures have reached up to 166 °F during summer months, well above the threshold for burn injuries to occur.^18^ This may place certain populations in this region with lower socioeconomic status (SES), such as homeless individuals, at higher risk of burn injuries and adverse burn injury outcomes.

Several studies have discussed the relationship of both individual-level and community-level indicators of SES and social disadvantage with the occurrence and outcomes of burn injuries.^15,20–27^ A Korean study found that more severe burn injuries were more frequent among lower SES groups.^20^ Another study found that lower SES individuals are predisposed to risk factors leading to a higher risk of burn injury, such as lower education level, lower income, and lack of employment.^25^ One specific United States study looking at burn patients who utilize methamphetamine was associated with lower SES, as well as a significantly greater TBSA and longer length of hospital stay.^28^ Historically, among minority racial groups, particularly Black and Latinx subgroups live in lower socioeconomic conditions, and individuals belonging to these populations have been shown to have a disproportionate burden of burn injuries and adverse outcomes as opposed to their White counterparts as well.^24,26,27,29^

Additional studies have demonstrated that patients with low SES, and specifically homeless individuals, may experience a higher TBSA in burn injuries than non-homeless patients—which may be due to their increased susceptibility to the risk factors associated with negative burn outcomes, such as alcohol consumption and assault.^21,22^ One US study further identified that homeless burn patients experienced more third-degree burns than non-homeless individuals.^30^ In addition to a greater TBSA and more severe burn thickness, another US study looking at homeless individuals specifically found that homeless burn patients are associated with longer lengths of hospital stay than their non-homeless counterparts.^31,32^ However, the evidence related to the association between burn injuries and SES is equivocal, as other studies have shown that there may not be a relationship between burn incidence and SES. A US-based study (2017) found no significant association between burn incidence and SES overall, although lower SES was associated with an increased burn injury risk in women.^23^ Another study reported that SES has no association with the rate of complications of burn injuries for patients, except for an increased mortality.^24^

Despite ample studies describing the impact of burn injuries on patients, there is still a lack of clarity on the impact of individual-level and community-level indicators of SES on burns, specifically in regard to burn etiology, hospital resource utilization, and mortality. Furthermore, there are minimal studies on this topic in the Southwestern United States, despite the temperatures of this region predisposing to increased incidence of certain types of burns.^12^ Therefore, we are filling this knowledge gap by conducting a comprehensive analysis of SES with burn injuries in Las Vegas, Nevada. To quantify SES, we uniquely utilized data from the 2017–2021 Distressed Community Index (DCI), a standardized tool that evaluates the economic well-being of United States communities at the zip code level.^33^ The DCI stratifies these zip codes into 5 quintiles ranging from prosperous to distressed. These quintiles were matched to the home addresses of patients hospitalized at a burn center in Las Vegas in order to determine the proportion of burn patients belonging to each group. Through this study, we primarily aim to investigate the association between burn injury etiologies with an individual-level and community-level index of SES, as well as provide evidence to support the development of infrastructure and other resources to mitigate this disparity.

## MATERIALS AND METHODS

### Study design and setting

This single-institution retrospective study collected data from all patients admitted to our American Burn Association verified burn center, located in Las Vegas, Nevada. The dates included in the data collection period were 5 years—from January 1, 2017 to December 31, 2021.

### Eligibility criteria

**Figure 1.**
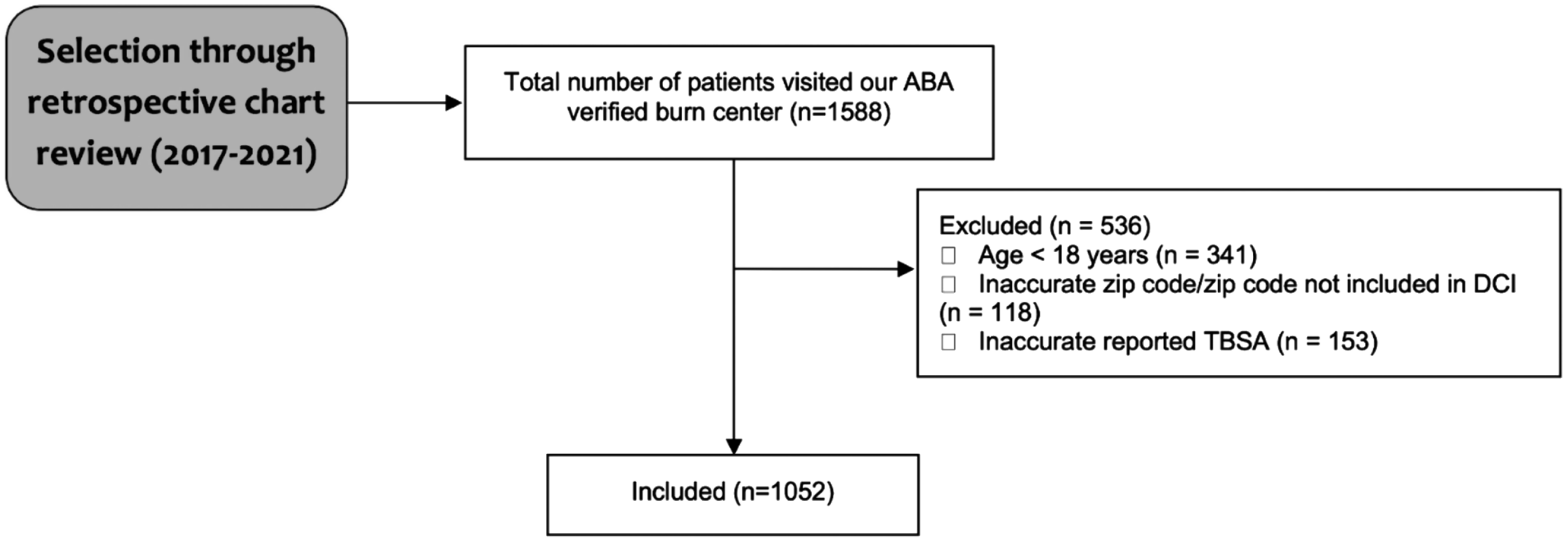
Selection Criteria Flowchart

A total of 1588 patients were admitted during the data collection period. All patients admitted during the data collection period were included. Patients were excluded if they were under 18 years old, their records contained an inaccurate zip code, a zip code not included in the DCI, or an inaccurate TBSA measurement (Figure 1). A total of 1052 patients met the inclusion criteria.

### Ethical considerations

This study was approved by our burn center’s Institutional Review Board on May 26, 2022 (UMC-2022-423) and was determined to involve no more than minimal risk. The data were de-identified and contained no personal identifiers. Furthermore, only the study investigators had access to the data.

### Data collection and variables

Data were gathered from our burn center’s patient registry. The data comprised both patient demographics (age, gender, race, ethnicity, insurance status, and residential zip code) and clinical variables, such as TBSA, burn severity, burn etiology, length of hospital stay, number of days on a ventilator, number of days in the Intensive Care Unit (ICU), total surgical interventions performed, and discharge disposition. At our burn center, TBSA is initially estimated using the Rule of Nines. Shortly after, the TBSA is reassessed by the burn nurses using the Lund Browder chart which is then reviewed and confirmed by the physician team. A table outlining the variables and measures of this study is shown in Table 1.

**Table 1.** Variables and Measures

Variable name	Raw variable description	Re-coded variable description	Measurement scale	Type
Age	Patient age	Patient age	Continuous	Numeric
Gender	Patient gender (male, female)	Patient gender (male, female)	Nominal	Categorical
Race	Patient identity as American Indian/Alaska Native, Asian, Black/African American, White, Native Hawaiian/Pacific Islander, Other Race, Unknown	Patient identity as Black, Asian, White, or Other Race	Nominal	Categorical
Ethnicity	Patient identity as Hispanic/Latino or not Hispanic/Latino	Patient identity as Hispanic/Latino or not Hispanic/Latino	Nominal	Categorical
Insurance status	Patient usage of Charity [i.e. Shriners] [Ret. 07-01-2020], Medicaid, Medicare, No Fault Automobile [Ret. 07-01-2020], Not Billed [For Any Reason], Other Government, Pending or Uncovered [Ret. 07-01-2020], Private/Commercial Insurance, Self Pay/Uninsured, Workers Compensation	Patient usage of public insurance, private insurance, worker compensation, or self-pay/uninsured	Nominal	Categorical
DCI quintiles	Patient residential address distress status (Prosperous, Comfortable, Mid-Tier, At- Risk, Distressed)	Patient residential address distress status (Prosperous, Comfortable, Mid-Tier, At- Risk, Distressed)	Nominal	Categorical
%TBSA	Total percentage of body surface area burned	Total percentage of body surface area burned	Continuous	Numeric
Burn degree	Severity of the burn injury (first, second, third)	Severity of the burn injury (1st, 2nd, 3rd)	Nominal	Categorical
Burn thickness	Depth of the burn injury (Partial or Full)	Depth of the burn injury (Partial or Full)	Nominal	Categorical
Burn etiology	Cause or origin of burn injury	Cause or origin of burn injury	Nominal	Categorical
Length of hospital stay	Number of days spent in the hospital	Number of days spent in the hospital	Continuous	Numeric
Ventilator use	Number of days spent using ventilator	Usage of a ventilator during hospital stay	Nominal	Categorical
ICU use	Number of days spent in the Intensive Care Unit. The ICU is a designated ward of a hospital for patients who require advanced respiratory, circulatory, and renal support or frequent neurological monitoring. ICU designation is decided by the burn physician based on the complexity of care that is required for the patient	Usage of the ICU during hospital stay	Nominal	Categorical
Surgical interventions	Number of surgical interventions performed on the patient	The presence of any surgical interventions performed on the patient	Nominal	Categorical
Discharge disposition	Status to which the patient is released/transferred after receiving medical care (Died in Hospital, Discharged Home [Prior Living Situation] with No Home Services, Discharged Home with Home Services, Discharged to Alternate Caregiver [Different Custody Prior to Admission] [Ret. 07-01-2020], Discharged to Foster Care [Ret. 07-01-2020], Discharged to Jail or Prison, Discharged to Street [Patient Without Home], Discharged/ Transferred to Long-Term Care Facility, Discharged/Transferred to Skilled Nursing Facility [SNF]/Nursing Home, Left Against Medical Advice or Discontinued Care, Transferred as Inpatient to Another Acute Burn Facility, Transferred as Inpatient to Another Hospital [Non-Burn] [Ret. 07-01-2020], Transferred to Hospice Care, Transferred to Inpatient Psychiatry Unit, Transferred to Inpatient Rehabilitation Facility)	Status to which the patient is released/transferred after receiving medical care (Died, Discharged Home, Transferred to another facility, Left against medical advice, Other)	Nominal	Categorical

ICU use: Received variable coded as length of stay in the ICU which was then dichotomized to Yes/No if the patient required care in the ICU.

### Statistical analysis

Data were first cleaned and re-coded for running analytical operations. All assumptions, including normality, and homogeneity of variance (through Levene’s test) were assessed. Categorical variables were represented as frequencies and proportions, whereas normally distributed continuous variables were represented by mean and standard deviations. The Chi-square/Fisher exact test was used for comparing the categorical groups. A post-hoc multiple *z*-tests to produce pairwise comparisons among all groups were used and *P* values were Bonferroni adjusted.^34^ Continuous outcomes among multiple groups were compared using one-way Analysis of variance (ANOVA) or Welch ANOVA if homogeneity of variance was not assumed. As one-way ANOVA is an omnibus test and only assesses the difference in means among 2 or more groups, however, it does not provide information on which groups are statistically different. Therefore, the Tukey’s post-hoc or Games Howell analysis (if the assumption of homogeneity of variance was violated) was also performed. The significance level was set at 0.05 and the normal approximation to the binomial distribution method was used to calculate 95% confidence intervals of proportions in the univariate analyses. All analyses were conducted using SPSS version 27 (SPSS, Chicago, IL, USA).

## RESULTS

### Demographics

A total of 1052 patients presented to our burn center during the study period. There were 746 male patients (70.9%) and 306 female patients (29.1%). The mean age was 52.01 ± 18.616 years. The proportion of patients, who resided in prosperous, comfortable, mid-tier, at-risk, and distressed communities were 15.3%, 16.3%, 7.0%, 23.0%, and 34.8%, respectively. Additional demographic data such as race, ethnicity, and insurance status are detailed in Table 2.

**Table 2.** Sociodemographic Characteristics (*n* = 1052)

Variable	Categories	*n* (%)	95% CI (LCL, UCL)
Age in years (M ± SD)	–	52.01 ± 18.616	50.89, 53.14
Gender	Female	306 (29.1)	26.3, 31.9
	Male	746 (70.9)	68.1, 73.6
Race	Asian	26 (2.5)	1.6, 3.6
	Black	211 (20.1)	17.6, 22.6
	White	652 (62.0)	58.9, 64.9
	Other Race	155 (14.7)	12.6, 17.0
Ethnicity	Hispanic or Latino	181 (17.2)	14.9, 19.6
	Not Hispanic or Latino	854 (81.2)	78.6, 83.5
Insurance	Public	689 (65.5)	62.5, 68.3
	Private	187 (17.8)	15.5, 20.2
	Worker compensation	102 (9.7)	7.9, 11.6
	Self-Pay/Uninsured	74 (7.0)	5.5, 8.7
Distressed Community Index (DCI) Quintiles	Prosperous	161 (15.3)	13.2, 17.6
	Comfortable	171 (16.3)	14.0, 18.6
	Mid-tier	74 (7.0)	5.5, 8.7
	At-risk	242 (23.0)	20.5, 25.7
	Distressed	404 (38.4)	35.5, 41.4

*All statistics are represented as frequencies and proportions unless stated otherwise. The percentage may not add up to 100% due to some missing data.*

*Abbreviations: CI, confidence interval; LCL, lower confidence limit; UCL, upper confidence limit; M, mean; SD: standard deviation*.

Table 3 illustrates the burn characteristics and hospital course of our cohort. The mean TBSA was 8.71%. Flame/contact was the most common burn mechanism (48.0%), followed by pavement (18.5%), scald (18.1%), chemical (8.1%), electrical (4.1%), and other (3.2%). 95.3% of our patients experienced second-degree burns/partial thickness, and 24.7% experienced third-degree or greater burns/full thickness. The mean length of hospital stay was 14.01 ± 23.893 days. During the hospital stay, 29.1% of patients required the use of the ICU, 18.6% of patients required the use of a ventilator, and 48.8% of patients required surgical intervention. Most of the patients (72.1%) were able to be discharged home.

**Table 3.** Burn Characteristics and Hospital Course (*n* = 1052)

Variable	Categories	*n* (%)	95% CI (LCL, UCL)
% TBSA (M ± SD)		8.71 ± 13.263	7.906, 9.511
Burn categories per % TBSA	<10% TBSA	791 (75.2)	72.4, 77.7
	10-20% TBSA	138 (13.1)	11.1, 15.3
	>20% TBSA	123 (11.7)	9.8, 13.7
Etiology of burns	Chemical	85 (8.1)	6.5, 9.8
	Electrical	43 (4.1)	2.9, 5.4
	Flame/Contact	505 (48.0)	44.9, 51.1
	Pavement	195 (18.5)	16.2, 21.0
	Scald	190 (18.1)	15.7, 20.5
	Other^a^	34 (3.2)	2.2, 4.4
Degree (second degree)	Yes	1003 (95.3)	93.8, 96.5
	No	49 (4.7)	3.5, 6.1
Degree (third degree/fourth degree)	Yes	260 (24.7)	22.1, 27.4
	No	792 (75.3)	72.5, 77.8
Length of Hospital Stay (M ± SD)		14.06 ± 23.893	12.61, 15.50
Intensive care unit (ICU) use	Yes	306 (29.1)	26.3, 31.9
	No	746 (70.9)	68.0, 73.6
Ventilator use	Yes	196 (18.6)	16.3, 21.1
	No	856 (81.4)	78.8, 83.6
Surgical interventions	Yes	513 (48.8)	45.7, 51.8
	No	539 (51.2)	48.1, 54.3
Discharge disposition	Died	62 (5.9)	4.5, 7.5
	Discharged Home	758 (72.1)	69.2, 74.7
	Transferred to another facility, including hospital, long-term care, inpatient facility	173 (16.4)	14.2, 18.8
	Left against medical advice	47 (4.5)	3.3, 5.9
	Other, including discharged to jail, discharged to street	12 (1.1)	0.6, 1.9

*All statistics are represented as frequencies and proportions unless stated otherwise. The percentage may not add up to 100% due to some missing data.*

^a^ *Other includes: Cold Injury, Frostbite, Other, Radiant Burn/Sun, Radiation*.

*Abbreviations: CI, confidence interval; LCL, lower confidence limit; UCL, upper confidence limit; M, mean; SD, standard deviation*.

**Figure 2.**
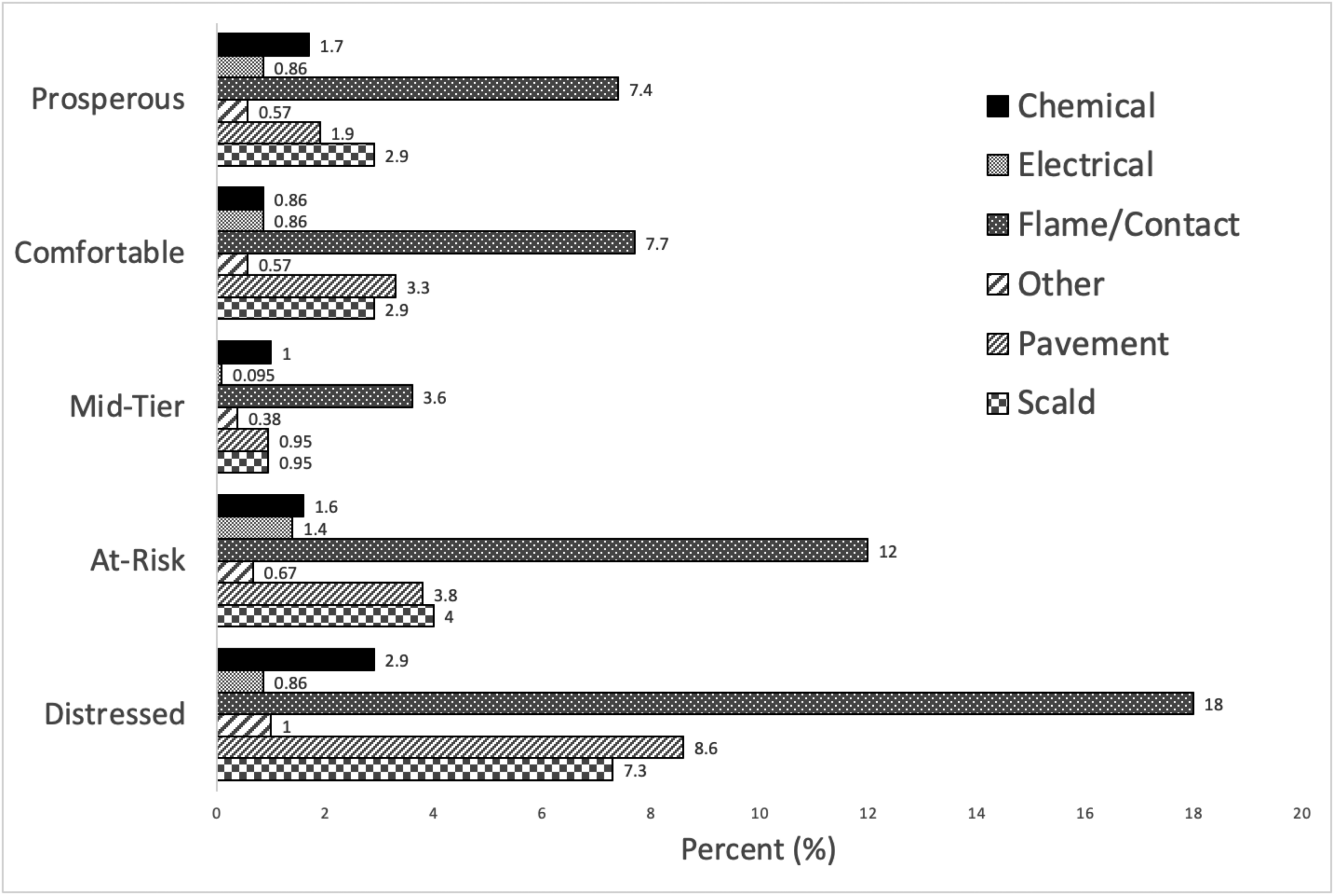
Percent Distribution of Burn Injury Etiology by Quintile

Patients residing in distressed communities contributed to the greatest number of burn injuries. These patients also experienced the greatest number of burn injuries per each burn mechanism with the exception of electrical burns (Figure 2). The distribution of burn injuries was not significant when compared to SES (*P* = 0.202). However, the distressed communities had a greater number of patients with public insurance (*P* < 0.001), a higher incidence of leaving against medical advice (*P* = 0.024), and a lesser number of patients being discharged home (*P* = 0.024).

### Burn etiology

Statistically significant differences were found when comparing the burn injury mechanism with age, gender, race, ethnicity, and insurance status (Table 4). Patients with chemical and electrical burn injuries were younger when compared to those with the pavement burns (*P* = 0.015, *P* = 0.020, Table 4). The rate of scald injuries (44.2%) was greatest among females whereas the rate of electrical burns (95.3%) was greatest among males (*P* < 0.001). White patients had a greater proportion of chemical (67.1%), flame/contact (66.9%), and pavement (63.6%) burns as compared to scald (46.8%) burns (*P* = 0.001). Patients who identified as Hispanic or Latino experienced a greater proportion of electrical burns (35.7%) as compared to flame/contact (15.4%) and pavement (14.5%) burns (*P* = 0.009). When examining insurance status, patients with public insurance experienced a greater proportion of pavement (82.6%) burns and patients with worker compensation experienced a greater proportion of electrical (53.5%) burns as compared to the other injury mechanisms (*P* < 0.001) (Supplementary Table S1).

**Table 4.** Comparing Sociodemographic Characteristics by Burn Etiologic Mechanisms (*n* = 1052)

Variable	Categories	Chemical	Electrical	Flame/contact	Pavement	Scald	Other^a^	*P* value
Age in years (M ± SD)	–	49.98 ± 23.12	47.58 ± 17.32	56.39 ± 21.70	58.98 ± 18.07	53.53 ± 22.99	58.15 ± 24.11	**0.001**
Gender	Female	7 (8.2)	2 (4.7)	149 (29.5)	55 (28.2)	84 (44.2)	9 (26.5)	**<0.001**
	Male	78 (91.89)	41 (95.3)	356 (70.5)	140 (71.8)	106 (55.8)	25 (73.5)	
Race	Asian	1(1.2)	0 (0.0)	10 (2.0)	6 (3.1)	8 (4.3)	1 (2.9)	**0.001**
	Black	11 (12.9)	4 (9.3)	96 (19.2)	42 (21.5)	51 (27.1)	7 (20.6)	
	White	57 (67.1)	29 (67.4)	334 (66.9)	124 (63.6)	88 (46.8)	20 (58.8)	
	Other Race	16 (18.8)	10 (23.3)	59 (11.8)	33 (11.8)	41(21.8)	6 (17.6)	
Ethnicity	Hispanic or Latino	16 (19.3)	15 (35.7)	76 (15.4)	28 (14.5)	41 (21.8)	5 (14.7)	**0.009**
	Not Hispanic or Latino	67 (80.7)	27 (64.3)	419 (84.6)	165 (85.5)	147 (78.2)	29 (85.3)	
Insurance	Public	52 (61.20)	7 (16.30)	331 (65.50)	161 (82.60)	116 (61.10)	22 (64.70)	**<0.001**
	Private	18 (21.20)	11 (25.60)	98 (19.40)	23 (11.80)	32 (16.80)	5 (14.70)	
	Worker Compensation	10 (11.80)	23 (53.50)	34 (6.70)	6 (3.10)	25 (13.20)	4 (11.80)	
	Self-Pay/Uninsured	5 (5.9)	2 (4.7)	42 (8.3)	5 (2.6)	17 (8.9)	3 (8.8)	
Distressed Community Index (DCI) Quintiles	Prosperous	18 (21.2)	9 (20.9)	78 (15.4)	20 (10.3)	30 (15.8)	6 (17.6)	0.090
	Comfortable	9 (10.6)	9 (20.9)	81 (16.0)	35 (17.9)	31 (16.3)	6 (17.6)	
	Mid-Tier	11 (12.9)	1 (2.3)	38 (7.5)	10 (5.1)	10 (5.3)	4 (11.8)	
	At-Risk	17 (20.0)	15 (34.9)	121 (24.0)	40 (20.5)	42 (22.1)	7 (20.6)	
	Distressed	30 (35.3)	9 (20.9)	187 (37.0)	90 (46.2)	77 (40.5)	11 (32.4)	

^a^ *Other includes: Cold Injury, Frostbite, Other, Radiant Burn/Sun, Radiation*. *% may not add up to 100% due to missing data*. *P values less than 0.05 are considered statistically significant.*

M, mean; SD, standard deviation.

### Healthcare resource utilization

Table 5 shows the clinical variables and hospital outcomes compared to the burn injury mechanism. The difference in mean TBSA between the burn mechanism groups was statistically significant (*P* < 0.001). Further analysis revealed that chemical burns differed significantly from electrical (*P* = 0.004), pavement (*P* = 0.029), scald (*P* = 0.021), and other burns (*P* = 0.022); electrical burns differed significantly from flame/contact burns (*P* = 0.015); flame/contact burns differed significantly from pavement (*P* < 0.001), scald (*P* < 0.001), and other burns (*P* < 0.001). Among full thickness burns, there was a greater proportion of flame/contact (28.5%) burns as compared to scald (18.4%) burns (*P* = 0.032). The mean length of hospital stay differed significantly between burn mechanisms (*P* = 0.002), with the greatest difference between pavement (17.59 ± 18.91 days) and scald (9.94 ± 15.72 days) burns. Patients who required stay in the ICU had a greater proportion of flame/contact (36.8%) burns than scald (13.2%) burns (*P* < 0.001). Additionally, those who required a ventilator during their hospital stay also had a greater proportion of flame/contact (26.1%) burns than scald (5.8%) burns (*P* < 0.001). In patients that required surgical intervention, pavement (56.9%) burns made up a greater proportion than electrical (25.6%) burns (*P* < 0.001). Finally, a greater proportion of patients with chemical (81.2%) burns were able to be discharged home as compared to pavement (55.4%) burns (*P* < 0.001).

**Table 5.** Comparing Clinical Characteristics and Hospital Outcomes by Burn Etiologic Mechanisms (*n* = 1052)

Variable	Categories	Chemical	Electrical	Flame/contact	Pavement	Scald	Other^a^	*P* value
%TBSA (M ± SD)	–	11.38 ± 15.96	4.26 ± 8.38	10.98 ± 16.30	6.23 ± 6.26	6.03 ± 7.55	3.12 ± 4.03	**<0.001**
Burn categories per % TBSA	<10% TBSA	54 (63.5)	40 (93.0)	351 (69.5)	157 (80.5)	157 (82.6)	32 (94.1)	**<0.001**
	10–20% TBSA	18 (21.2)	1 (2.3)	67 (13.3)	28 (14.4)	23 (12.1)	1 (2.9)	
	>20% TBSA	13 (15.3)	2 (4.7)	87 (17.2)	10 (5.1)	10 (5.3)	1 (2.9)	
Partial thickness burn	No	4 (4.7)	1 (2.3)	20 (4.0)	15 (7.7)	7 (3.7)	2 (5.9)	0.34
	Yes	81 (95.3)	42 (97.7)	485 (96.0)	180 (92.3)	183 (96.3)	32 (94.1)	
Full thickness burn	No	70 (82.4)	32 (74.4)	361 (71.5)	145 (74.4)	155 (81.6)	29 (85.3)	**0.032**
Length of Hospital Stay (M ± SD)		11.07 ± 16.58	7.49 ± 16.92	15.70 ± 29.39	17.59 ± 18.91	9.94 ± 15.72	8.26 ± 8.76	**0.002**
ICU use	No	60 (70.6)	33 (76.7)	319 (63.2)	139 (71.3)	165 (86.8)	30 (88.2)	**<0.001**
	Yes	25 (29.4)	10 (23.3)	186 (36.8)	56 (28.7)	25 (13.2)	4 (11.8)	
Ventilator use	No	70 (82.4)	40 (93.0)	373 (73.9)	161 (82.6)	179 (94.2)	33 (97.1)	**<0.001**
	Yes	15 (17.6)	3 (7.0)	132 (26.1)	34 (17.4)	11 (5.8)	1 (2.9)	
Surgical interventions	No	49 (57.6)	32 (74.4)	249 (49.3)	84 (43.1)	101 (53.2)	24 (70.6)	**0.001**
	Yes	36 (42.4)	11 (25.6)	256 (50.7)	111 (56.9)	89 (46.8)	10 (29.4)	
Discharge disposition	Died	4 (4.7)	0 (0.0)	44 (8.7)	14 (7.2)	0 (0.0)	0 (0.0)	**<0.001**
	Discharged home	69 (81.2)	39 (90.7)	359 (71.1)	108 (55.4)	163 (85.8)	20 (58.8)	
	Transferred to another facility, including hospital, long-term care, inpatient facility	5 (5.9)	3 (7.0)	78 (15.4)	60 (30.8)	16 (8.4)	11 (32.4)	
	Left against medical advice	4 (4.7)	1 (2.3)	19 (3.8)	11 (5.6)	10 (5.3)	2 (5.9)	
	Other, including discharged to jail, discharged to street	3 (3.5)	0 (0.0)	5 (1.0)	2 (1.0)	1 (0.5)	1 (2.9)	

*% may not add up to 100% due to missing data*. *P values less than 0.05 are considered statistically significant*.

^a^ *Other includes: Cold Injury, Frostbite, Other, Radiant Burn/Sun, Radiation*.

*M, mean; SD, standard deviation*.

## DISCUSSION

The main objective of this study was to examine the relationship of individual-level and community-level SES indicators with burn etiologies and hospital courses by utilizing the DCI to classify patient SES by zip code. This study is unique in that it utilizes a community-level indicator of SES to identify the occurrence of burn injuries in different SES groups, specifically in the Southwestern United States. By exploring burn experiences in different groups, interventions can be put in place to develop resources to decrease burn incidence and ameliorate burn disparities.

Overall, it was found that SES did not come to significance in regards to burn incidence. However, the most distressed quintile according to the DCI were found to have the greatest number of burns overall, as well as the greatest number of burns per every etiology except electrical burns. In addition, different burn etiologies were found to be associated with different demographic characteristics as well as hospital courses and outcomes.

The primary outcome of interest of this study was the occurrence of burn injuries based on an individual’s SES as indicated by the DCI. However, it was found that the distribution of burn injury patients was not significant when utilizing the DCI to quantify SES. Other papers found primarily contradictory information, finding associations between burns and SES as well as burns and factors integral to SES.^20,24,25^ One of these nationwide studies found that severity-based incidence of burn injuries was significantly correlated with SES, as well as the most severe burn injuries in lower SES groups.^20^ Our study specifically quantified SES via community-level indices, however other studies analyzing similar subjects utilized different modes of SES classification. These studies primarily utilized individual-level indices, such as quantifying SES based on a household basis from monthly salary, owned property data, and insurance status.^20,24^ This likely contributes to how our results differ from other studies analyzing similar patient populations.

Beyond study design, there are multiple potential explanations as to why no significance was found. Because there is high diversity in population in Las Vegas and a wide range of SES throughout different Las Vegas zip codes, this may have prevented the DCI from being the most accurate representation of SES due to a lack of individualized SES characterization. Another plausible explanation is that burns affect different populations relatively equally. One study analyzing the intersection of SES and gender with burn injuries corresponded with our findings, stating that lower SES did not significantly increase the risk of burn injury overall.^23^ However, it is worth noting that this study similarly utilized zip code of residence and US census data to standardize SES, further elucidating that different methods of quantifying SES may significantly impact results.

While the more distressed communities did not show an association with SES, patients residing in distressed communities contributed to the greatest number of burn injuries overall and the greatest number of burns per burn etiology, with the exception of electrical burns (Figure 2). Additionally, these communities were associated with a higher incidence of leaving against medical advice and a lower incidence of being discharged home (Table 5). These results align with other studies demonstrating that populations with a lower SES are associated with worse burn outcomes, such as higher mortality rates.^24^ These results further suggest the presence of a relationship between SES and burn incidence, and warrant future studies regarding this topic.

Despite the lack of significance identified when analyzing burns with SES, there were additional findings shown to be significant when looking at demographic features most associated with burn incidence. In regard to burn etiology, it was found that electrical burns were associated with younger populations and pavement burns with older populations (Table 4). This finding is corroborated in another study indicating that the age group most associated with electrical burns is 25–34 year olds.^35^ This may potentially be explained by work-related accidents resulting in electrical burns in new young workers.^36^ Furthermore, females were associated with scald burns whereas males had a greater incidence of electrical burns (Table 4). Other studies have supported these findings on a global scale, showing a significant correlation between electrical burns with males and scald burns with females.^37–39^

Interestingly, this study showed a high number of pavement burns in comparison to other studies on similar topics.^20,24,25^ Pavement burns have been associated geographically with the Southwestern United States, including the Las Vegas area, specifically in summer months.^18,40^ It was noted that pavement burns were associated with homelessness, as well as neuropathy and other medical conditions predisposing patients to seizures or syncope. The proportion of individuals with homelessness status in Las Vegas is estimated to be 24.6 per 10 000 people, which is substantially higher than the United States national average of 18 out of every 10 000 people.^41^ The pavement temperatures in Las Vegas can reach up to 166 °F in summer months, and homeless patients may have a lack of accessibility to shade and other resources to prevent pavement burns.^12,18^

White patients were shown to make up the greatest amount of burns in every burn category (Table 4). This corresponds with other studies stating that White males statistically being the largest burn patient population in the United States.^27^ Yet, previous studies additionally describe that marginalized racial groups may be disproportionately affected by burn injuries and are correlated with worse burn outcomes.^24,26,27^ One United States study found that African American males had a higher risk of mortality from burn injuries than other racial groups, consistent with other studies describing health inequities in black patients.^27^ Another study explained that African American burn patients were associated with increased ventilator days, longer LOS, and higher rates of septicemia.^24^ Further research is needed to assess the intersectionality of race with other demographic dimensions such as SES in relation to burn incidence (Supplementary Table S2). Hispanic and Latino populations were associated with a higher amount of electrical burns (Table 4). This may be due to job sectors associated with higher concentrations of this demographic group in Las Vegas. For example, in the United States in 2023, 27.7% of construction workers, a field susceptible to electrical burns, were found to be Hispanic, despite only 19.1% of the US population being Hispanic.^42,43^

Interestingly, insurance status was associated with different burn etiologies. Public insurance users were associated with an increased incidence of pavement burn, whereas individuals utilizing worker’s insurance were associated with electrical burns (Table 4). More distressed populations and homeless individuals were associated with public insurance, and these individuals may have an increased likelihood of lacking resources to protect from pavement burn injuries.^19,22^ Furthermore, electrical burns are associated with workplace injuries, in which individuals may utilize their employer’s insurance.^44^ Increased research must be conducted to further explore the relationship of demographic characteristics with burn incidence.

Beyond demographic factors, different burn etiologies were additionally associated with varying hospital courses and outcomes. Pavement burns were shown to have a significantly longer LOS and less discharge compared to scald and chemical burns (Table 5). Pavement burns have been associated with more severe burn injuries than other types of burns, and our findings corroborate other studies demonstrating that pavement burns are correlated with longer hospital stays and more operative intervention than other burn etiologies.^45^ The pavement in Las Vegas is capable of causing significant burns instantaneously or in little time, resulting in rapid onset of severe burns and complications requiring intensive care.^18^ Furthermore, pavement burn patients were more likely to be homeless, potentially implicating a lack of a safe discharge disposition. In regard to surgical interventions, pavement burns were found to require a higher amount of surgeries than electrical burns (Table 5). Research has shown that pavement burns often require multiple debridement procedures, whereas electrical burns may only require EKG and lab monitoring to prevent severe complications.^40,44,45^ It was additionally found that flame and contact burns tended to have a higher rate of ICU and ventilator utilization than scald burns (Table 5). Flame and contact burns also have been shown to yield higher degrees of extensive physiological damage than other burn etiologies, with infections and respiratory distress as common complications.^46,47^

As demonstrated by our data and previous studies, burn injuries have the potential to cause widespread bodily damage to patients. This study emphasizes the importance of implementing novel resources to prevent burn injuries in order to improve patient outcomes. Yet, there are already various studies that have discussed implemented or proposed public health interventions targeted toward decreasing burn incidence and ameliorating burn disparities. Many of the resources available that were identified focused on the assessment and prevention of pediatric burn injuries.^48–50^ Many of these studies focused on parental education specifically to promote burn prevention in their children. An Iranian study focused on providing educational content to mothers of children aged 1–3 on burn injury prevention to promote the development of home safety.^50^ Other studies focused on catering to adult populations more susceptible to burn injury. One 2023 study created a fire safety manual and held training sessions for homeless encampment residents. Researchers identified that tent fires were found to be common among homeless individuals in colder months, and they provided equipment and developed escape strategies with these populations that would allow for avoidance of burn injuries.^51^ Another study set in New York City focused uniquely on elderly burn prevention education.^52^ This educational programming was provided to elderly adults that are a part of a community-based senior center, and featured content on burn risk factors, home safety and methods of prevention. A majority of participants reported learning new information from these programs, emphasizing the importance of providing these resources to vulnerable populations.^49^

Through using these studies as foundation, new resources may be developed and existing resources can be innovated to continue combatting burn injuries and their ramifications. Although there is ample literature documenting strategies toward pediatric burn prevention, there has been minimal documentation of interventions targeting adult burn victims, such as work-related burn prevention resources. For work-related burn injuries specifically, creating more educational training resources for employees on utilizing equipment appropriately and how to respond to a burn emergency, as well as implementing more rigorous safety protocol and hazardous equipment maintenance are possible opportunities to prevent burn occurrence and negative outcomes. Furthermore, for certain etiologies of burns such as pavement burns, there is a lack of preventive resources potentially due to the decreased incidence of these burns. While educational resources for these types of burns may be beneficial in burn prevention, development of new public infrastructure to provide shade for individuals or even providing complimentary shoes or sandals at clinics and community centers for homeless individuals could also uniquely play a role in burn prevention for pavement burn injuries. In areas such as the Southwestern United States, these resources may be especially beneficial.

This study provides valuable insight into the incidence of burns based on SES. A major strength of this study is that it utilizes the DCI to standardize SES. This is the first community-level indicator of SES, allowing for a unique analysis of burn patients and novel insights as to how community-level classification may impact these findings. Additionally, to our knowledge, this is the first study looking at this topic in the Southwest region of the United States, as this geographic area is prone to different etiologies of burns.

However, this study has several limitations that may have impacted the results acquired. Due to the retrospective and cross-sectional nature of this study, a cause-and-effect relationship was not able to be established from the data collected. This study was additionally single-institution, limiting external validity. This study may not have been truly representative of the burn incidences in Las Vegas, and there may have been a lack of samples from specific zip codes. In addition, the selection bias of excluding specific patient groups (patients under 18, patients with inaccurate TBSA, and patients with inaccurate zip codes) may have altered the results of this study by preventing a more generalizable conclusion (Figure 1).

Furthermore, various TBSA calculation methodologies may be used across institutions. Variance in the interpretation of TBSA among physicians may lead to misclassification bias and may impact generalizability of these findings. Finally, there was exclusion of patients in this study without an accurate zip code for SES classification, which may have primarily impacted the inclusion of homeless individuals in this study. Because of this, there may have been exclusion of severe burn experiences in individuals with low SES.

## CONCLUSION

This study displays the results of a cross-sectional study in Las Vegas, Nevada regarding the incidence of burn injuries in relation to SES indicated by patients’ zip code of residence. In addition to SES, we discuss the role of various demographic factors as well as burn etiology in relation to individuals’ burn outcomes. There was found to not be a significant association between SES and burn incidence, which may be due to a number of factors, including the diversity of SES within zip codes in Las Vegas. It may be beneficial for future studies to utilize multiple standardized measurements of burn severity and SES to determine the relationship between these variables. Future studies may also look to expand data collection to include variables such as comorbidities, occupation, housing conditions, education level, behaviors like smoking and alcohol use, and other psychosocial stressors to better understand the multifactorial nature of burn injuries. Additionally, controlling for these variables in regression analysis may provide comprehensive prediction models that can assess one’s risk of burn injury. We also propose future studies to conduct a propensity-matched analysis to minimize the selection bias in observational studies. Because burn injuries are a public health emergency, these studies may contribute toward the advancement of resources promoting burn awareness and prevention programs for vulnerable communities.

## SUPPLEMENTARY MATERIAL

Supplementary material is available at *Journal of Burn Care & Research* online.

irae131_suppl_Supplementary_Materials
