# Supplementary material for: The Individual-Level and Community-Level Social Determinants of Burn Injuries: A Single-Institution Study From the Southwestern United States
Source: J Burn Care Res. 2024 Jul 6;46(2):307–17. doi: 10.1093/jbcr/irae131 (PMC11879727; doi:10.1093/jbcr/irae131)
Supplement: irae131_suppl_Supplementary_Materials [file irae131_suppl_supplementary_materials.docx]

**Supplementary Material**

**Table 1.** Comparison of Combined DCI Quintiles by Burn Etiologic Mechanism (n=1,052)

| **Variable** | **Categories** | **Chemical** | **Electrical** | **Flame/Contact** | **Pavement** | **Scald** | **Other*** | **P value** |
| --- | --- | --- | --- | --- | --- | --- | --- | --- |
| **Combined Community Index Quintiles** | Prosperous/Comfortable | 27 (31.8) | 18 (41.9) | 159 (31.5) | 55 (28.2) | 61 (32.1) | 12 (35.3) | 0.220 |
|  | Mid-Tier | 11 (12.9) | 1 (2.3) | 38 (7.5) | 10 (5.1) | 10 (5.3) | 4 (11.8) |  |
|  | At-risk/  Distressed | 47 (55.3) | 24 (55.8) | 308 (61.0) | 130 (66.7) | 119 (62.6) | 18 (52.9) |  |

** Other includes: Cold Injury, Frostbite, Other, Radiant Burn/Sun, Radiation*

**Table 2.** Intersectional Analysis (n=1,044)

|  | | **White (n = 652)** | | | | | **Non-White (n = 392)** | | | | |
| --- | --- | --- | --- | --- | --- | --- | --- | --- | --- | --- | --- |
| **Variable** | **Categories** | **Male**  **Hispanic or Latino** | **Male**  **Not Hispanic or Latino** | **Female**  **Hispanic or Latino** | **Female**  **Not Hispanic or Latino** | **P value** | **Male**  **Hispanic or Latino** | **Male**  **Not Hispanic or Latino** | **Female**  **Hispanic or Latino** | **Female**  **Not Hispanic or Latino** | **P value** |
| **Etiology of Burns** | **Chemical** | 6 (10.5%) | 47 (11.2%) | 0 (0%) | 4 (2.7%) | **<0.001** | 10 (13%) | 13 (7.3%) | 0 (0%) | 3 (2.9%) | 0.002 |
|  | **Electrical** | 8 (14%) | 20 (4.8%) | 0 (0%) | 1 (0.7%) |  | 7 (9.1%) | 6 (3.4%) | 0 (0%) | 0 (0%) |  |
|  | **Flame/**  **Contact** | 24 (42.1%) | 214 (50.8%) | 14 (63.6%) | 80 (53.7%) |  | 26 (33.8%) | 85 (47.8%) | 12 (48%) | 39 (37.5%) |  |
|  | **Pavement** | 11 (19.3%) | 81 (19.2%) | 1 (4.5%) | 30 (20.1%) |  | 13 (16.9%) | 33 (18.5%) | 3 (12%) | 21 (20.2%) |  |
|  | **Scald** | 7 (12.3%) | 46 (10.9%) | 7 (31.8%) | 28 (18.8%) |  | 18 (23.4%) | 33 (18.5%) | 9 (36%) | 39 (37.5%) |  |
|  | **Other*** | 1 (1.8%) | 13 (3.1%) | 0 (0%) | 6 (4%) |  | 3 (3.9%) | 8 (4.5%) | 1 (4%) | 2 (1.9%) |  |
| **Length of Hospital Stay (M±SD)** |  | 10±13.1 | 13.7±22.7 | 9.3±8.7 | 15.9±23.3 |  | 17.9±44.2 | 12.1±17.8 | 16.7±22.7 | 14.6±21 |  |
| **ICU use** | **Yes** | 24 (42.1%) | 128 (30.4%) | 5 (22.7%) | 49 (32.9%) | 0.252 | 18 (23.4%) | 40 (22.5%) | 5 (20%) | 28 (26.9%) | 0.816 |
|  | **No** | 33 (57.9%) | 293 (69.6%) | 17 (77.3%) | 100 (67.1%) |  | 59 (76.6%) | 138 (77.5%) | 20 (80%) | 76 (73.1%) |  |
| **Surgical Intervention** | **Yes** | 26 (45.6%) | 199 (47.3%) | 15 (68.3%) | 72 (48.3%) | 0.282 | 39 (50.6%) | 83 (46.6%) | 15 (60%) | 52 (50%) | 0.631 |
|  | **No** | 31 (54.4%) | 222 (52.7%) | 7 (31.8%) | 77 (51.7%) |  | 38 (49.4%) | 95 (53.4%) | 10 (40%) | 52 (50%) |  |
| **Ventilator use** | **Yes** | 14 (24.6%) | 89 (21.1%) | 1 (4.5%) | 35 (23.5%) | 0.216 | 10 (13%) | 21 (11.8%) | 3 (12%) | 18 (17.3%) | 0.616 |
|  | **No** | 43 (75.4%) | 332 (78.9%) | 21 (95.5%) | 114 (76.5%) |  | 67 (87%) | 157 (88.2%) | 22 (88%) | 86 (82.7%) |  |
| **Discharge disposition** | **Died** | 3 (5.3%) | 23 (5.5%) | 1 (4.5%) | 14 (9.4%) | 0.010 | 2 (2.6%) | 6 (3.4%) | 2 (8%) | 8 (7.7%) | 0.060 |
|  | **Discharged Home** | 44 (77.2%) | 296 (70.3%) | 21 (95.5%) | 96 (64.4%) |  | 67 (87%) | 126 (70.8%) | 22 (88%) | 74 (71.2%) |  |
|  | **Transferred to another facility, including hospital, long-term care, in-patient facility** | 8 (14%) | 79 (18.8%) | 0 (0%) | 33 (22.1%) |  | 5 (6.5%) | 28 (15.7%) | 0 (0%) | 17 (16.3%) |  |
|  | **Left against medical advice** | 2 (3.5%) | 22 (5.2%) | 0 (0%) | 2 (1.3%) |  | 3 (3.9%) | 12 (6.7%) | 1 (4%) | 4 (3.8%) |  |
|  | **Other, including discharged to jail, discharged to street** | 0 (0%) | 1 (0.2%) | 0 (0%) | 4 (2.7%) |  | 0 (0%) | 6 (3.4%) | 0 (0%) | 1 (1%) |  |

** Other includes: Cold Injury, Frostbite, Other, Radiant Burn/Sun, Radiation*
